# Frailty is associated with objectively assessed sedentary behaviour patterns in older adults: Evidence from the Toledo Study for Healthy Aging (TSHA)

**DOI:** 10.1371/journal.pone.0183911

**Published:** 2017-09-11

**Authors:** Borja del Pozo-Cruz, Asier Mañas, María Martín-García, Jorge Marín-Puyalto, Francisco J. García-García, Leocadio Rodriguez-Mañas, Amelia Guadalupe-Grau, Ignacio Ara

**Affiliations:** 1 Department of Exercise Sciences, University of Auckland, Auckland, New Zealand; 2 GENUD Toledo Research Group, University of Castilla-La Mancha, Toledo, Spain; 3 CIBER of Frailty and Healthy Aging (CIBERFES), Madrid, Spain; 4 GENUD Research Group, Faculty of Health and Sport Sciences, University of Zaragoza, Huesca, Spain; 5 Geriatric Department, Hospital Virgen del Valle, Toledo, Spain; 6 Geriatric Department, Hospital Universitario de Getafe, Getafe, Spain; 7 ImFINE Research Group, Department of Health and Human Performance, Technical University of Madrid, Madrid, Spain; Nathan S Kline Institute, UNITED STATES

## Abstract

**Objective:**

The aim of this study was to examine the association of sedentary behaviour patterns with frailty in older people.

**Setting:**

Clinical setting.

**Design:**

Cross-sectional, observational study.

**Participants and measurements:**

A triaxial accelerometer was used in a subsample from the Toledo Study for Healthy Aging (519 participants, 67–97 years) to assess several sedentary behaviour patterns including sedentary time per day, the number and duration (min) of breaks in sedentary time per day, and the proportion of the day spent in sedentary bouts of 10 minutes or more. Frailty was assessed using the Frailty Trait Scale (FTS). Regression analysis was used to ascertain the associations between sedentary behaviour patterns and frailty.

**Results:**

Sedentary time per day and the proportion of the day spent in sedentary bouts of 10 minutes or more, were positively associated with frailty in the study sample. Conversely, the time spent in breaks in sedentary time was negatively associated with frailty.

**Conclusion:**

In summary, breaking up sedentary time and time spent in sedentary behaviour are associated with frailty in older people.

## Introduction

Frailty in older adults is considered a biological condition where poor resolution of several physiological systems to maintain homoeostasis occurs after a low-power stressor event [1, 2]. Frailty is associated with alterations in the musculoskeletal, vascular and central nervous systems [3]. Prevalence of frailty in Spain is 8.4%, with an additional prevalence of pre-frailty of 41.8%, therefore approximately 50% of people over 65 years are categorized as frail or pre-frail [4]. Socio-economic costs associated with frailty are also of relevance [5]. Frailty has been associated with an increase in hospitalization rates, falls, incident disability, decreased mobility, and higher mortality rates [2, 6, 7]. In the last decade, frailty has been recognized as one of the most promising indicators to help prevent disability [8]. Finding mechanisms to prevent frailty are therefore of interest.

Sedentary behaviours, including those characterized by low energy expenditure while in a sitting or reclining posture, have been shown to contribute to adverse outcomes. Even in the absence of other risk factors, sedentary behaviour has recently emerged as an independent cardiovascular risk factor [9–11] and is related to all causes of mortality in a dose-response manner [12], possibly because sedentary behaviour influences homeostasis and function of many if not all body systems [13]. Lack of exercise and a sedentary lifestyle is one of the most significant public health problems of the 21st century [14], the effect that a sedentary lifestyle exerts on frailty is poorly assessed [1].

Da Silva Coqueiro et al. [15] recently reported an association between sedentary behaviour as assessed by a questionnaire and frailty status among adults over 60 years. Similarly, objectively assessed sedentary behaviour was associated with frailty among adults over 50 years old in the National Health and Nutrition Examination Survey [16] independent of moderate-to-vigorous physical activity (MVPA). Sedentary behaviour patterns (i.e. how sedentary behaviour is accumulated) can make an impact on the wider health of individuals [17]. For example, an increased number of bouts of sedentary behaviour per day is associated with worse health outcomes including reduced cardiovascular health or physical function [17]. Inserting bouts of activity into otherwise sedentary time (ST) is associated with better physical function in older adults [18]. Breaking up sedentary time has also been positively associated with a lower risk of disability in the activities of daily life and inversely associated with impairments and physical dependence in older age, independent of MVPA [19]. Hence, reducing or breaking up long sedentary periods seems to be a feasible and promising approach expected to attenuate the consequences of frailty among older adults. Nonetheless, there is a lack of data regarding the relationship between sedentary behaviour patterns and frailty in this population group [1]. Therefore, the aim of this study was to examine the associations of various sedentary behaviour patterns with frailty in older people.

## Methods

### Research design and participants

Data were taken from the Toledo Study for Healthy Aging (TSHA), whose complete methodology has been reported elsewhere [4, 20]. Briefly, the TSHA is a population prospective cohort study aimed at studying the determinants and consequences of frailty in institutionalised and community-dwelling individuals older than 65 years living in the province of Toledo, Spain. A subsample of 626 volunteers was assessed on sedentary behaviour patterns. From these, 107 were excluded due to incomplete or invalid accelerometer data. A total of 519 participants were finally included. All the subjects gave their informed consent in written and the study was performed in accordance with the Helsinki Declaration of 1975, as last modified in 2000, regarding the conduct of clinical research, and was approved by the Ethical Committee of the Toledo Hospital (CEIC).

### Outcome measures

#### Frailty

The Frailty Trait Scale (FTS) was used to assess frailty [21]. Briefly, the FTS includes 7 dimensions (i.e. energy balance and nutrition, activity, nervous system, vascular system, weakness, endurance, and slowness) operationalised through 12 items. Each item represents a biological trait. All items but one (“chair test”, which scored 0 [worst status] to -5 [best status]) are scored 0 (best status) to 4 (worst status). The total score was calculated by adding all the scores in each item divided by total score possible for each individual, multiplied by 100. The total score ranged from 0 (best score) to 100 (worst score).

#### Sedentary behaviour patterns

Sedentary behaviour patterns were assessed by accelerometry (ActiGraph, ActiTrainer 3X, Fort Walton Beach, FL). Accelerometer output is an activity count, which is the weighted sum of the number of accelerations measured over a time period or epoch (a 1 minute epoch was used in this study). The intensity of activity is assessed from the weighted sums, which are proportional to the magnitude of measured acceleration. Participants were asked to wear the accelerometer on their left hip for 7 consecutive days. Sleeping periods were removed from the analyses and considered non-wear time. Moreover, periods of at least 60 consecutive minutes of zero counts were also considered as non-wear time and removed as well [22]. Only data from participants with 4 or more valid days of accelerometer data (i.e. a day with 480 minutes or more of wear time) were included in the analyses (n = 519).

Each minute with less than 100 counts was considered sedentary time [23, 24]. Time per day (min) spent in sedentary time was then registered. A break in sedentary time (BST) was defined as at least 1 min where the accelerometer registers ≥100 counts following a sedentary period. The number and duration (min) per day of BST were recorded. A 10-min bout of sedentary time (ST-10) was defined as a period of at least 10 consecutive minutes where the accelerometer registered <100 counts/min. The number, duration (min) and proportion over total 10-min bouts per day of ST-10 were recorded. All outcomes were weighted by daily averaged wear time on valid days (i.e. outcome summed over all wear time divided by the number of successfully monitored days for each participant). The different sedentary pattern variables were standardized (*Z*-score = [observed—sample mean]/sample SD). In an effort to account for the combined effect of both duration and number of the different patterns, two composite Z-scores representing patterns of ST [ST-COMP = Z-score ST-10 (number/day) + Z-score ST-10 (minutes/day)] and patterns of BST [BST-COMP = Z-score BST (number/day) + Z-score BST (minutes/day)] were then computed.

#### Adherence to WHO physical activity guidelines

In order to analyse the adherence of physical activity (PA) in the study with recommendations for public health proposed by the World Health Organization, the accumulation of at least 150 min/week of moderate physical activity or 75 min/week of vigorous physical activity or as an equivalent combination of moderate and vigorous physical activity was assessed with an accelerometer [25].

#### Anthropometrics and confounding variables

Height was measured to the nearest millimetre using a portable stadiometer (Medizintechnik seit 1890, KaWe, Germany), and weight was measured with a SECA precision scale (SECA 884 floor scale, Germany). Individuals removed their shoes, socks and heavy clothes prior to weighing. Body mass index (BMI) was calculated as weight (kg) divided by height squared (m^2^). A number of confounders were assessed. Mental status was assessed using the Minimental Scale questionnaire [26]. Comorbidity history was assessed using the Charlson Comorbidity index [27]. Physical performance was assessed by means of the Short Physical Performance Battery [28]. MVPA was assessed by accelerometry (i.e. each minute of 1952 or more counts was considered MVPA) [23]. Current medication (i.e. number of drugs), age, and gender were also recorded.

### Statistical analysis

Data were analysed using PASW Statistic, version 23.0.0, with statistical significance set at p<0.05 (two-tailed). Descriptive statistics (mean ± SD) were calculated for all outcome measurements of the study.

Multiple linear regressions were used to examine the associations between frailty and the different sedentary behaviour patterns assessed. A model adjusted by age, gender, comorbidity status, mental health, and polypharmacy status was fitted for each of the sedentary behaviour pattern outcomes.

Participants were clustered into 2 different groups according to the adherence to WHO PA guidelines status (i.e. meeting or not the guidelines) and were compared using t-test for independent measurements. Participants within each of the former groups were re-allocated to either less BST group (i.e. group falling below the 50th percentile of BST) or more BST group (i.e. group falling over the 50th percentile of BST) and then compared using t-test for independent measurements.

## Results

Characteristics of the participants are shown in Table 1. A total of 519 older adults aged 67–97 including 234 males and 285 females, were included in the study.

**Table 1:** Characteristics of the participants in the study (n = 519).

Variables	
Age (yr)	78.84 (4.55)
Gender, male (%)	45.10
Body Mass Index (kg/m^2^)	30.54 (4.74)
Charlson Comorbidity Index (0–31)	1.12 (1.56)
Mini Mental State Examination (0–30)	23.01 (4.40)
Polypharmacy status (%)	
<3	19.10
3–4	26.60
>5	53.90
Educational Status (%)	
Never attended to school	41.10
Less than primary school	41.10
At least primary school	17.40
Short Physical Performance Battery (0–12)	8.44 (2.26)
Accelerometer data	
Average wear time per valid day (min)	780.70 (84.68)
MVPA (min/day)	17.62 (21.87)
LPA (min/day)	223.03 (91.69)
ST (min/day)	540.04 (93.87)
10-min bouts of ST (number/day)	16.09 (3.46)
10-min bouts of ST (min/day)	494.29 (114.98)
10-min bouts of ST (percentage) (%)	68.37 (14.48)
BST (number/day)	69.17 (19.28)
BST (min/day)	240.66 (99.39)
BST (number/hour)	5.29 (1.29)
BST (min/hour)	18.30 (6.91)
Meeting WHO PA guidelines, yes (%)	29.60
Frailty trait scale (0–100)	39.84 (15.12)

All values are mean (SD) unless otherwise stated. All accelerometer variables are adjusted for wear time; ST: Sedentary time; LPA: Light physical activity; MVPA: Moderate-to-vigorous physical activity; BST: Breaks in sedentary time; PA: physical activity; Meeting WHO PA guidelines: accumulating 150 min/week of moderate physical activity or 75 min/week of vigorous physical activity or equivalent combination of moderate and vigorous physical activity.

Table 2 shows the results of different multiple regressions analyses testing the associations between various sedentary behaviour patterns and frailty in the TSHA study. After adjusting for several covariates (i.e. age, gender, comorbidity status, mental health, and polypharmacy status) ST (β, 95CI% = 0.015, 0.004 to 0.027; *p* = 0.03), ST-10 (proportion) (β, 95CI% = 0.079, 0.234 to 0.195; *p* = 0.04), BST (minutes/day) (β, 95CI% = -0.031, -0.048 to -0.014; *p* = 0.03), and BST-COMP (β, 95CI% = -0.805, -1.339to -0.210; *p* = 0.01), were significantly associated with frailty in the study sample. However, ST-10 (number/day and minutes/day), BST (number/day), and ST-COMP were not significantly associated with frailty.

**Table 2:** Linear regression analysis for the association of various sedentary behaviour patterns with frailty in the TSHA study.

Predictors of interest								
Models, β (95%CI)	ST (minutes/day)	ST-10 (number/day)	ST-10 (minutes/day)	ST-10 (proportion)	BST (number/day)	BST (minutes/day)	ST-COMP^a^	BST-COMP^b^
Model^†^	0.015 (0.004 to 0.027)*	0.044 (-0.371 to 0.459)	0.007 (-0.006 to 0.021)	0.157 (0.079 to 0.234)*	-0.065 (-0.148 to 0.018)	-0.031 (-0.048 to -0.014)*	0.220 (-0.386 to 0.829)	-0.805 (-1.339 to -0.210)*
R^2^	0.224	0.223	0.225	0.238	0.227	-0.232	0.224	0.234

^†^Model, β (95%CI): Adjusted for age, gender, comorbidity status (Charlson index), mental health (Mini Mental Scale), and polypharmacy status

ST: Sedentary time; ST-10: 10-min bouts of ST; BST: Breaks in sedentary time; ST-COMP: ST Composite score; BST-COMP: BST Composite score

All predictors of interest were adjusted for wear time

^a^Sum of ST-10 (minutes/day) and ST-10 (number/day) z-scores

^b^Sum of BST (number/day) and BST (minutes/day) z-scores

*Significant at *p*<0.05

**Fig 1:**
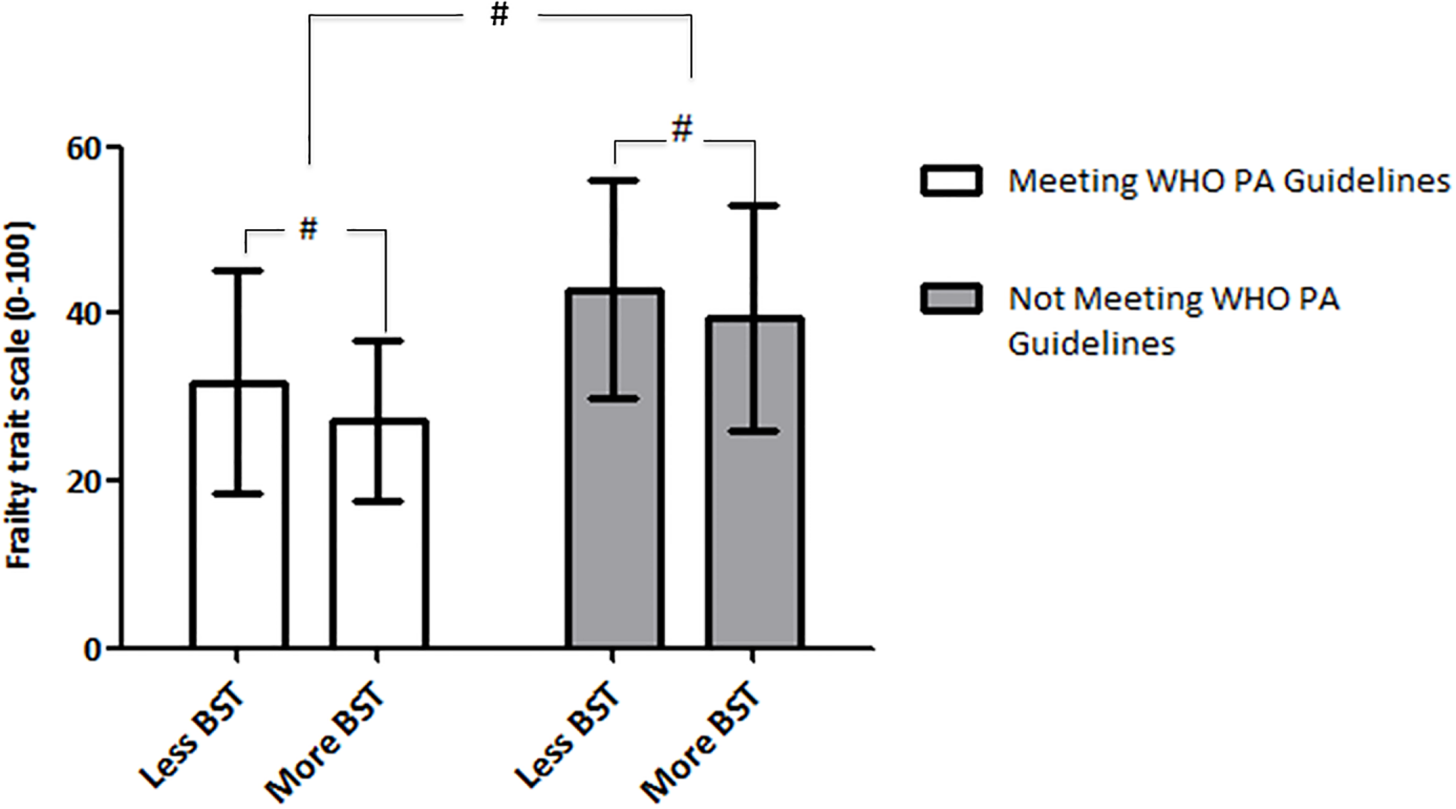
Frailty trait scale score in different groups. BST: breaks in sedentary time (number/hour); WHO; World Health Organization; PA: physical activity; Meeting WHO PA guidelines: the accumulation of at least 150 min/week of moderate physical activity or 75 min/week of vigorous physical activity or equivalent combination of moderate and vigorous physical activity; More BST: BST≥P50 (P50 = 4.47); Less BST: BST<P50; #: p<0.05.

Participants who met the WHO PA guidelines scored statistically significant less in the Frailty Trait Scale than those who did not meet the PA guidelines (Fig 1). In both groups, those who fell into the bottom half of interruptions of sedentary time had a statistically significant higher frailty level than those depicting more BST (*p*<0.05) (Fig 1).

## Discussion

To our knowledge, this is the first study that comprehensively analyses the impact of objectively assessed sedentary patterns beyond total time spent in sedentary behaviour on frailty in older adults. The main findings were that in adjusted models (i.e. in models adjusted by relevant demographic and medical confounders) frailty was associated with sedentary time per day, the proportion of the day spent in sedentary bouts of 10 minutes or more, and time spent in breaks of sedentary time. These results may shed some light on the ongoing discussion regarding the health consequences of sedentary lifestyles and could generate novel hypotheses that could help in informing future public health interventions in order to prevent frailty among older adults.

Available evidence suggests that, regardless of MVPA, spending time in sedentary activities increases the odds of being frail among older adults [15, 16]. Our results confirm and extend those of Da Silva Coqueiro et al. [15] and Blodgett et al. [16] (i.e. frailty was associated with ST in our sample), but also verified thatthe proportion of the day spent in sedentary bouts of 10 minutes or more is a more powerful predictor of frailty than raw sedentary time or sedentary time spent in 10 min blocks. The former opens the hypothesis that health consequences of sedentary behaviour may be connected to the display of other behaviours. Compositional analysis of time spent in different behaviours is therefore required to fully understand the impact sedentary behaviour may have on health, including frailty among older adults.

In an attempt to understand more precisely how the pattern of accumulation of sedentary time may have an impact on frailty scores in older adults, we combined the number and duration of bouts of 10 minutes or more spent in sedentary time into a novel sedentary time compositional score. Previous work has shown an association between sedentary behaviour and frailty status among adults and older adults [15, 16]. Similarly, in order to reflect the potential combined implication of both duration and number of BST on frailty, a compositional score from the number of and minutes spent in breaks of sedentary time was created (BST-COMP). Previous work has demonstrated that BST is associated with physical function and disability in older adults [18, 19]. Our empirical work extends and confirms the hypothesis that interrupting ST has the potential of enhancing the wider health of individuals by demonstrating that not only the number of BST but also duration of those BST may have an impact on frailty status among older individuals. Collectively, the results from our study support and extend to frailty the inactivity physiology hypothesis. Future experimental research is warranted to clarify the potential mechanisms underpinning these associations.

From a public health perspective, reducing sedentary behaviour and engaging in light physical activity, for instance, by inserting short bouts of activity into otherwise sedentary periods, may be a more feasible and less challenging approach for older adults than taking part in more strenuous activities in order to promote health [29]. Our findings reveal that having fewer breaks in sedentary periods was associated with higher frailty level among the study sample. The reverse is also true. This is of interest, as only a minor proportion of older adults meet the WHO PA recommendations (30% in our sample). Therefore, while efforts on MVPA promotion should be sustained, guidelines for older adults should also reinforce the idea of breaking up ST more often in order to prevent frailty among this population group.

Key strengths of the study include the relatively large sample, the objective measures of sedentary behaviour patterns, and the inclusion of a novel analytical approach by deriving new variables that reflects more accurately sedentary behaviour patterns and therefore provide unique knowledge in the field with potential clinical relevance. The cross-sectional nature of the research design used does not allow definitive conclusions to be drawn around the causal relationship between the variables of the study. There are some inherent issues with sedentary behaviour patterns being derived from accelerometers, such as the use of <100 counts/minute as a threshold to determine sedentary activities [30] or the use of 1-min epoch length that may impact the generalization of the results [31]. Despite these limitations, our findings contribute to the current literature and ongoing discussion on the impact of sedentary behaviour on frailty among older adults. More research is warranted around the potential effects of activity insertion of different intensities to prevent frailty in this population group. Moreover, longitudinal experimental designs are necessary to overcome some of the research-design inherent limitations of this study and confirm the results showed here.

In conclusion, sedentary time, time spent in more active behaviour, and daily proportion of time spent in sedentary behaviour bouts of 10 minutes or more are associated with frailty in older people in the TSHA study. Altogether, these results may point to the pathways through which engaging in frequent, short bouts of activity insertions into otherwise sedentary periods can attenuate frailty among older adults. Our results suggest that interventions should therefore not only be focused on reducing the time spent in sedentary activities but also on how that time is accrued in order to prevent frailty in older people. However, longitudinal, experimental research, preferably in form of RCT, is required to confirm the causality of the relationships observed in the current study.
